# Towards comprehensive and effective strategies to address sexual health

**DOI:** 10.1186/s13584-017-0171-2

**Published:** 2017-08-24

**Authors:** Charmaine Gauci, Natasha Azzopardi-Muscat

**Affiliations:** University of Malta Faculty of Health Sciences, Tal-Qroqq, Msida, Malta

**Keywords:** Sexual health, Strategy, Israel, HIV, STIs

## Abstract

Sexual health is an important global public health concern. Planning effective strategies to improve sexual health requires a high degree of attention to the local epidemiological trends and cultural context where the strategy is to be implemented. The paper by Chemtob et al. describes the process to develop a plan that aims to reduce the burden of Sexually Transmitted Infections in Israel by 2025. This commentary argues that increased attention to planning and implementation of sexual health policy is required in order to address the real burden of disease. Sexual health should not be merely addressed from a communicable disease control perspective but should comprehensively address health and wellbeing of all population groups through a positive approach in line with the WHO current definition of sexual health. As even traditionally culturally conservative societies are experiencing rapid changes in attitudes and practices towards sexual lifestyles, the challenge is to ensure that sexual health strategies combine evidence-informed measures and good practices with culturally appropriate communication and implementation approaches.

## Introduction

The paper by Chemtob and colleagues [1] “A national strategic plan for reducing the burden of sexually transmitted infections in Israel by the year 2025”, is a valuable contribution to the literature on policy making in the important area of sexual health. In general terms, the paper describes the approach undertaken to address sexual health in Israel. The authors provide a useful framework for those seeking to develop policy in this somewhat challenging field. They approach their needs assessment through a comprehensive combination of methods including local data, comparative international data, evidence on what works and engaging with expert opinion leaders. The latter point, which is often overlooked, is critical to obtain the political and cultural views on a topic which is often normatively and emotionally laden. Being a relatively small country, public health researchers and practitioners are able to exploit the proximity to policy makers and this can add an important dimension to ensure acceptability of the final proposals.

The authors clearly demonstrate the large variation in reported epidemiology and practice that exists between countries in Europe pointing to the need for a more robust evidence base for policy making in the area of sexual health. They rightly highlight the issue of underreporting which may be a key factor explaining the degree of variation observed and reach the conclusion that different approaches to better capture the real epidemiological situation are necessary.

## Discussion

The paper focusses primarily on estimation of the burden of STIs, screening for STIs and implementation of strategies to reduce the burden of STIs.

- Estimation of the burden of STIs

Various countries have different systems of collection of data. This is essential for each individual country to have a good picture of trends in order to have a clear picture of the situation including the risk groups to be targeted as well as monitoring of progress being made. Robust and reliable surveillance systems are critical in order to inform relevant stakeholders in an accurate and timely fashion. The epidemiology of STIs including HIV is constantly evolving and information on new cases, routes of transmission and patient outcomes are important needed to monitor the epidemiology of infection. Surveillance systems therefore need to keep pace with the changing nature of epidemics at national and European levels.

There are various forms of surveillance systems for STIs which include:

- Passive surveillance, which collects information from laboratories, clinics and STI control programmes and service
- Behavioural surveillance collects data from behaviours of specific target groups
- Sentinel which identify specific centres and they report on their practice eg that seen in Germany
- Periodic cross sectional surveys

In many countries, the number of reported STIs represent only the “tip of the iceberg” for various reasons. The commonest reason is because most infections are asymptomatic or else they are not diagnosed. There could also be unreported symptomatic cases due to stigma and discrimination, fear of potential conflict with sexual partner especially in the married group, self-prescription of medicines from pharmacies or empirical treatment in the community. Many countries have legal frameworks which mandate notification by doctors to notify cases of infectious diseases. Some countries also have a legal obligation for the notification of positive samples from laboratories [2].

Underreporting for STIs can be tackled through the development of a sentinel surveillance system focusing on a selected primary care doctors who will report on the cases they see in their practice and this is extrapolated to the general population. Enhanced surveillance of STI in Europe is essential to outline the distribution of disease and evaluate the public health response to control the transmission of infections [3]. The European Centre for Disease Prevention and Control collates data across Europe and supports countries in Europe to ensure that surveillance data are of high quality. This information is essential to inform on policy development.

- Screening of STIs

Screening individuals to determine if they have been infected with or exposed to an infectious disease is a core public health strategy. Early identification of cases and early linkage to treatment has important public health benefits. In the area of STIs, early treatment reduces the risks of transmission to others and also reduces the risks to health of the patient including long term effects such as infertility. Early diagnosis for HIV and linkage to care through a treat all approach is one of the UN targets for ending HIV/ AIDS. Routine, voluntary HIV testing benefits both affected individuals and their intimate partners by facilitating early access to prevention, care and treatment services.

- Implementation of strategies to reduce the burden of STIs

A comprehensive approach to disease prevention, testing and care is a basic public health strategy. The European Centre for Disease Prevention and Control commissioned a project with the aim of increasing the understanding of how HIV and STI can be prevented among vulnerable groups in the broader context of sexual health and health promotion [4]. The main outcomes of this project focused on the need to promote more synergy between European bodies and NGOs with regard to data collection, reporting and publication. This could be facilitated though the development of a template or toolkit, to aid countries in gathering comparable data on a variety of sexual health indicators and outcomes of public health interest. Projects on specific vulnerable groups, such as migrants or MSM, need to be brought together. International collaboration could be strengthened to develop initiatives focusing on STI/HIV prevention in the broader context of sexual health and health promotion among risk groups. This report also highlights the fact that good practices are often presented and shared however these need to be evaluated to identify areas for improvement.

This paper by Chemtob et al. outlines the components of the Israeli strategy to control STIs. The strategy covered the main aspects which are evidence based. One crucial element in strategy formulation is the identification of vulnerable groups based on the national epidemiology of the people being affected. Combination STI and HIV prevention measures are more cost effective if taken through a comprehensive approach.

The focus on social determinants of sexual health is unfortunately a missing element in many strategies. Yet social determinants have an impact on health of vulnerable populations. Such groups may have barriers such as preventing high risk individuals from accessing services offering preventive services. These barriers include national legislation such as laws governing sexual conduct. One of these laws includes age of sexual consent which affects the age of first start of sexual encounters. Sex between same gender sex is now legal in several countries and so no legal barriers are identified in this area. Accessibility of service provision and fear of stigma and marginalisation are other reasons which may keep persons from coming forward to access services.

This paper has a second important contribution. It raises the issue of different epidemiological patterns between religious groups. As societies in Europe and elsewhere become more ethnically heterogenous due to increased migration, the way in which these demographic changes affect sexual lifestyles needs to be taken into account in planning sexual health policy.

The authors place a specific emphasis on groups at risk such as men who have sex with men (MSM) and commercial sex workers. However, the wider implications of changing sexual attitudes and practices amongst the general population are somewhat overlooked. The importance of regular population surveys complemented by in-depth qualitative research with specific groups needs to be given more importance. The value of the numbers and trends of STIs achieved through passive and active surveillance needs to be placed in the broader context and narrative of societal attitudes and practices. These are often difficult to extract and confront in conservative and religious countries. Yet, evidence points towards rapid societal change leading to fast changing sexual health practices. These are often least well addressed by those countries not typically used to dealing with such a situation and the Israeli approach is to be commended in this regard.

In 2016 the World Health Organisation published its strategy on Sexually Transmitted Infections (STIs) for the period 2016–2021 [5]. This is based on the aspiration that everybody has free and easy access to sexually transmitted infection prevention and treatment services, that no new infections, no sexually transmitted infection-related complications and deaths, and zero discrimination. The strategy is linked to sectoral strategies on HIV and Hepatitis and is based on the framework of the SDGs. Unfortunately, trends in STIs including resistant gonorrhoea currently make the prospect of achieving this vision rather bleak.

However if each country takes steps to define the specific populations that are most affected by sexually transmitted infection epidemics including populations most likely to have a high number of sex partners, such as sex workers and their clients as well as other populations such as men who have sex with men, transgendered people, and people with an existing sexually transmitted infection, including people living with HIV this is a good start. Other groups considered to be particularly vulnerable to sexually transmitted infections include young people and adolescents, women, mobile populations, children and young people living on the street, prisoners, drug users and people affected by conflict and civil unrest.

## Conclusions

In our view, the key element to ensure success in addressing sexual health at country level is that the response is one which is firmly rooted in epidemiological evidence but which gives due attention to the social context. Planning and implementing sexual health strategies is a challenging policy area for public health. The rising trends in STIs combined with rapidly changing societal norms and sexual lifestyles and practices make the need to address sexual health at both individual and population levels an urgent one. Public health professionals and policy makers need to use the whole array of health promotion tools ranging from legislation, effective communication, empowerment of individuals and communities as well as strengthening health services in order to make positive progress to improve sexual health and well-being.
